# Supplementary material for: Optical Spectroscopic Detection of Mitochondrial Biomarkers (FMN and NADH) for Hypothermic Oxygenated Machine Perfusion: A Comparative Study in Different Perfusion Media
Source: Sensors (Basel). 2025 Jun 28;25(13):4031. doi: 10.3390/s25134031 (PMC12251590; doi:10.3390/s25134031)
Supplement: Supplementary file 1 [file sensors-25-04031-s001.zip › sensors-3676421-supplementary.pdf]

Optical Spectroscopic Detection of Mitochondrial Biomarkers (FMN and NADH) for Hypothermic Oxygenated Machine Perfusion: A Comparative Study in Different Perfusion Media

Lorenzo Agostino Cadinu [1,2,*], Keyue Sun [2], Chunbao Jiao [2], Rebecca Panconesi [2], Sangeeta Satish [2,3], Fatma Selin Yildirim [2], Omer Faruk Karakaya [2], Chase J. Wehrle [3], Geofia Shaina Crasta [2], Fernanda Walsh Fernandes [2], Nasim Eshraghi [2], Koki Takase [2], Hiroshi Horie [2], Pier Carlo Ricci [4], Davide Bagnoli [5], Mauricio Flores Carvalho [6], Andrea Schlegel [2,3] and Massimo Barbaro [1,*]

1 Department of Electric and Electronic Engineering, University of Cagliari, Via Marengo 2, 09123 Cagliari, Italy
2 Department of Inflammation & Immunity, Lerner Research Institute, Cleveland Clinic, Cleveland, OH 44195, USA; sunk2@ccf.org (K.S.); jiaoc@ccf.org (C.J.); satishs2@ccf.org (S.S.); yildirf@ccf.org (F.S.Y.); karakao@ccf.org (O.F.K.); crastag2@ccf.org (G.S.C.); eshragn@ccf.org (N.E.); takasek@ccf.org (K.T.); horieh@ccf.org (H.H.); schlega4@ccf.org (A.S.)
3 Transplantation Center, Cleveland Clinic, Cleveland, OH 44195, USA; wehrlec@ccf.org
4 Department of Physics, University of Cagliari, Complesso Universitario di Monserrato, S.P. Monserrato-Sestu Km 0, 700, 09042 Monserrato, Italy; carlo.ricci@dsf.unica.it
5 Medica Spa, Via Della Beverara, 46/d, 40131 Bologna, Italy; davide.bagnoli@medica-spa.com
6 Bridge to Life Ltd., Atlanta, GA 30096, USA; m.carvalho@b2ll.com
* Correspondence: lorenzoa.cadinu@unica.it (L.A.C.); massimo.barbaro@unica.it (M.B.)

Section S1. Composition of the perfusion solution

The chemical composition of perfusion solutions utilized in our experiments is reported in Table S1 indicates the most widespread use of the perfusion solutions considered.

Table S1a. Components of Belzer MPS UW

CONSTITUENT	AMOUNT/1000 ML
**Adenine (free base)**	0.68 g
**Calcium Chloride (dihydrate)**	0.068 g
**Dextrose (+)**	1.80 g
**Glutathione (reduced)**	0.92 g
**HEPES (free acid)**	2.38 g
**Hydroxyethyl Starch**	50.0 g
**Magnesium Gluconate**	1.13 g
**Mannitol**	5.4 g
**Potassium Phosphate (monobasic)**	3.4 g
**Ribose, D(-)**	0.75 g
**Sodium Gluconate**	17.45 g
**Sodium Hydroxide**	0.70 g
**Water To 1000 mL Volume**	-

Table S1b. Components of Custodiol

CONSTITUENT	AMOUNT/1000 ML
**Sodium Chloride**	0.8766 g
**Potassium Chloride**	0.6710 g
**Potassium hydrogen 2-Ketoglutarate**	0.1842 g
**Magnesium Chloride 6 H20**	0.8132 g
**Histidine HCI H20**	3.7733 g
**Histidine**	27.9289 g
**Tryptophan**	0.4085 g
**Mannitol**	5.4651 g
**Calcium Chloride**	0.0022 g
**Water To 1000 mL Volume**	-

Table S1c. Components of Celsior

CONSTITUENT	AMOUNT/1000 ML
**Glutathione**	0.921 g
**Mannitol**	10.930 g
**Lactobionic acid**	28.664 g
**Glutamic acid**	2.942 g
**Sodium hydroxide**	4.000 g
**Calcium chloride dihydrate**	0.037 g
**Potassium chloride**	1.118 g
**Magnesium chloride hexahydrate**	2.642 g
**Histidine**	4.650 g
**Water To 1000 mL Volume**	-

Table S1d. Components of IGL-1

CONSTITUENT	AMOUNT/1000 ML
**Lactobionic acid**	35,8 g
**Adenosine**	1,336 g
**Allopurinol**	0,136 g
**Glutathione**	0,922 g
**Polyethylene glycol (PEG 35000)**	1 g
**Potassium dihydrogen phosphate**	3,402 g
**Raffinose pentahydrate**	17,84 g
**Magnesium sulfate heptahydrate**	1,232 g
**Sodium Hydroxide: qs pH: 7.4**	-
**Water To 1000 mL Volume**	-

Table S1e. Utilization of perfusion solution

Preservation medium	Organ
**Belzer**	Liver, kidney
**Celsior**	Liver, kidney
**Custodiol HTK**	Heart
**IGL-1**	Liver, kidney

Section S2. Stock solution preparation

Stock solutions of flavin mononucleotide (FMN) were prepared by accurately weighing 60 mg of FMN powder using a Metter AE 163 microbalance. Due to the hygroscopic nature of FMN, the powder was pre-dried by heating at 45 °C for 15 minutes to ensure precision in measurement. This drying and weighing process was repeated until a constant weight was achieved. The dried FMN powder was then transferred to a 1000 ml Type A volumetric flask and dissolved in a 0.9% saline solution (B. Braun Melsungen AG, Germany). The flask was immediately covered with foil and stored in a dark refrigerator at 5 °C. To ensure thorough dissolution, the solution was mixed continuously for a minimum of 5 hours. The final concentration of the FMN solution was 0.06 mg mL^{-1}.

For the preparation of NADH solution, 500 mg of NADH powder, which is non-hygroscopic, was weighed without the need for drying. The powder was placed in a 50 ml Type A volumetric flask, and saline solution was added to the meniscus level. This resulted in a final NADH concentration of 10 mg mL^{-1}.

Section S3. Perfusion solution preparation

C.1 Belzer Solution Preparation

Initially, 50 grams of hydroxyethyl starch were weighed and dissolved in 500 mL of water heated to a temperature range of 38-42 °C in a 1000 mL glass beaker. Given the propensity of hydroxyethyl starch to form aggregates that are difficult to dissolve, heating the water was essential to facilitate dissolution. The solution was vigorously mixed, and aggregates were manually broken up using a glass rod to ensure complete dissolution. Additionally, an ultrasonic bath (Bandelin Sonorex) was employed to further aid in the dissolution of sugars and salts. Subsequently, each component was individually weighed and added to the solution. The resulting mixture was then transferred to a 1000 mL Type A volumetric flask. The pH of the solution was measured using a pH meter (pHep5, HI98128 Hanna Instruments) and adjusted by adding sodium hydroxide. Finally, water was added to reach a total volume of 1000 mL.

C.2 Preparation of Other Solutions

The preparation of the other solutions was less demanding as they did not contain critical components like hydroxyethyl starch. However, our objective was to study the spectrophotometric behavior of the components rather than their clinical applications. Consequently, we did not ensure the sterility of the perfusion solution. To inhibit the growth of bacteria and mold, the solution was prepared and stored in a refrigerator at 5 °C for a few hours before being used for sample preparation and measurements. An ultrasonic bath (Bandelin Sonorex) was employed when necessary to aid in the dissolution of sugars and salts. The procedure involved dissolving all components in 500 mL of

water heated to 35-39 °C. The solution was then transferred into a 1000 mL volumetric flask, with additional water added to reach the meniscus level.

Section S4. Measurement procedure

To prevent contamination of the previous solution during spectrophotometric measurements, a meticulous cuvette washing protocol was implemented. Since highly diluted solutions were used, it was essential to avoid any cross-contamination between samples. After each measurement, the cuvette was washed with a portion of the solution being analyzed, which was then discarded. This washing procedure ensured the elimination of any residual contamination, thereby enhancing the reliability of the results. The use of 50 mL flasks provided an ample quantity of sample solution for effective cuvette washing after each measurement. For sample injection into the cuvette, a different glass or plastic Pasteur pipette was utilized for each sample. Additionally, the quartz cuvette was thoroughly cleaned and transparent by first washing it with water, followed by acetone or isopropyl alcohol to remove any water traces, and finally drying it with a common hairdryer. This comprehensive cleaning procedure is schematically depicted in Figure S1. The software of the Jasco spectrophotometer and spectrofluorometer allows for data export in CSV format. After exporting, the data was imported and analyzed using Excel, Python 3.11 in Spyder environment, version 5.4.3 and OriginPro® 2018 for visualization and spectral analysis.

Prior to performing spectrometric measurements, the cuvette was rinsed by flushing it three times with the same sample solution taken from the volumetric flask. On the fourth rinse, the cuvette was filled with the sample solution for the actual measurement. This procedure ensured that no contamination from previous samples affected the results. Each washing portion was discarded after use, maintaining the integrity of the measurements.

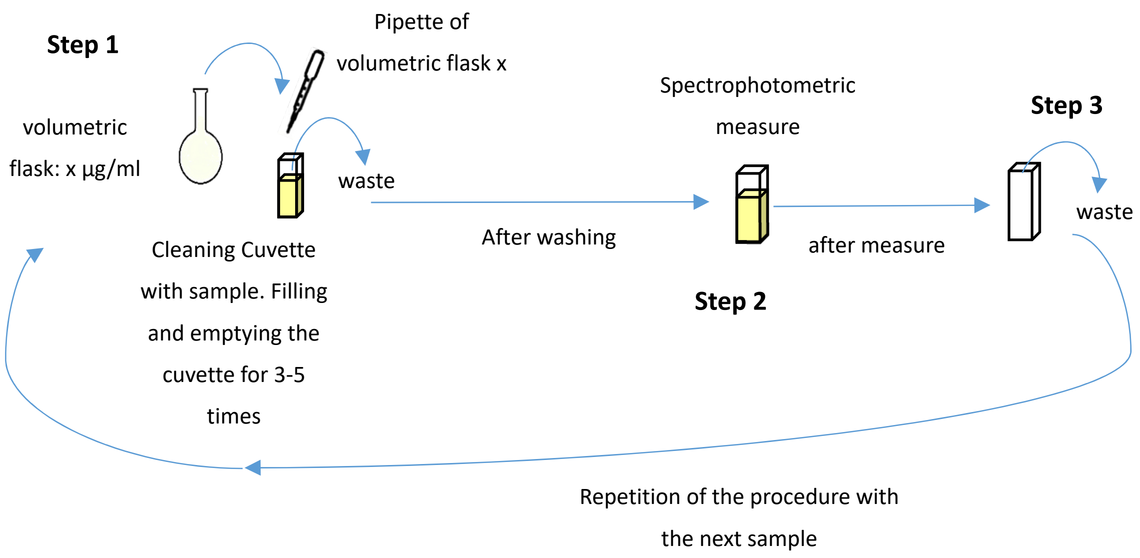

Figure S1. Schematization of measurement procedure

Characteristics of the measurement conducted with the V-750 Double Beam UV-Visible

Spectrophotometer by Jasco are reported in Table S2a:

Table S2a. Parameters of Spectrophotometer

Measurement range	200 – 800 nm
Data Interval	0.5 nm
Bandwidth	2.0 nm
Response	0.06 nm
Scan mode	Continuous
Scan speed	400 nm/min

Section S5. Absorption and fluorescence spectra of perfusion media

The perfusion media studied in these experiments absorb in the blue region (440-490 nm) to varying extents. Belzer solution exhibits higher absorbance than Celsior and Custodiol solutions, indicating that Belzer interferes with FMN more significantly at the studied FMN concentration (Figure 7). Consequently, it is expected that the fluorescence intensity for the same excitation wavelength will be higher in Belzer solution compared to the other media.

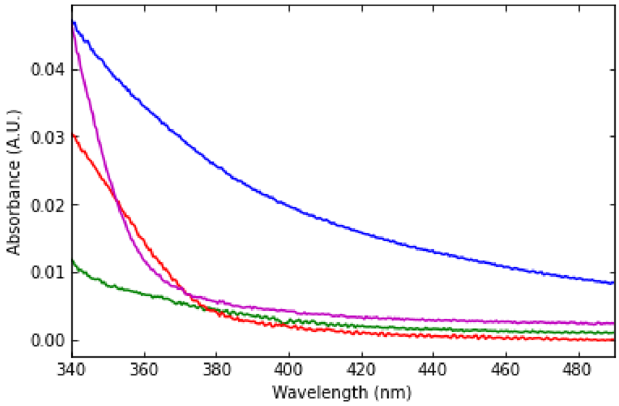

Figure S2. Absorption of perfusion in near ultraviolet and blue region

The absorption of the perfusion media increases significantly in the near-ultraviolet region, especially below 350 nm. These aqueous solutions are primarily composed of sugars, organic and inorganic salts, amino acids, nitrogenous bases, starches, and buffering agents. With increased excitation energy intensity, a greater number of chemical bonds can be excited. In the ultraviolet region, photon energy is sufficiently high to cause electronic transitions of electrons in many chemical bonds within the molecules, resulting in increased absorbance in this region. Photon energy in the visible light range is generally lower and insufficient to excite electrons in many types of chemical bonds of molecules dissolved in the solution. As a result, fewer bonds are excited, leading to lower recorded absorbance. Figure S2 clearly demonstrates these phenomena.

The high total absorbance is also due to the high concentration of various solutes in the solution; indeed, the total solute concentration is 84.678 g/L in Belzer, 55.956 g/L in Celsior, and 40.123 g/L in Custodiol.

In Figure S3, 3D and 2D plots of the spectra of the perfusion media are presented. An excitation range of 300-490 nm, corresponding to the absorption range of FMN, was selected to investigate potential emissions in the yellow region (500-600 nm) and determine their intensity.

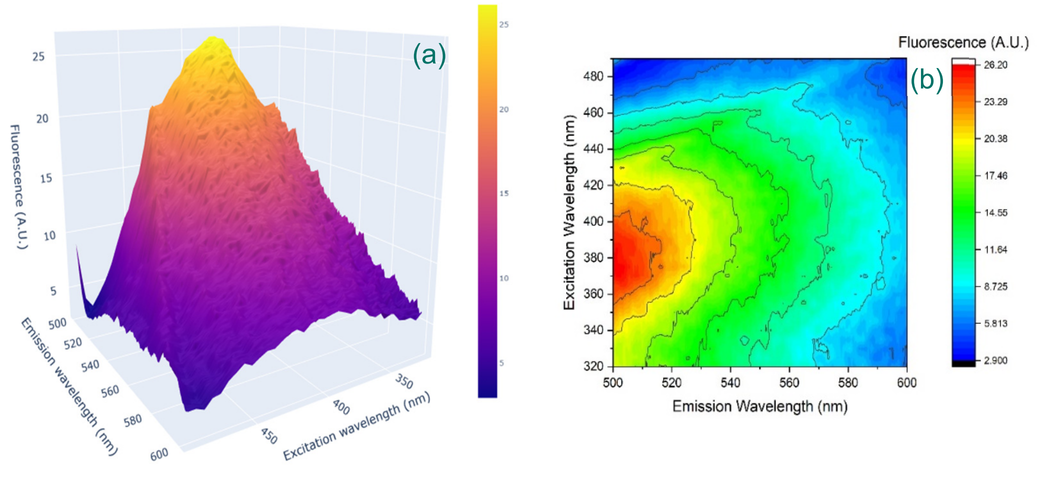

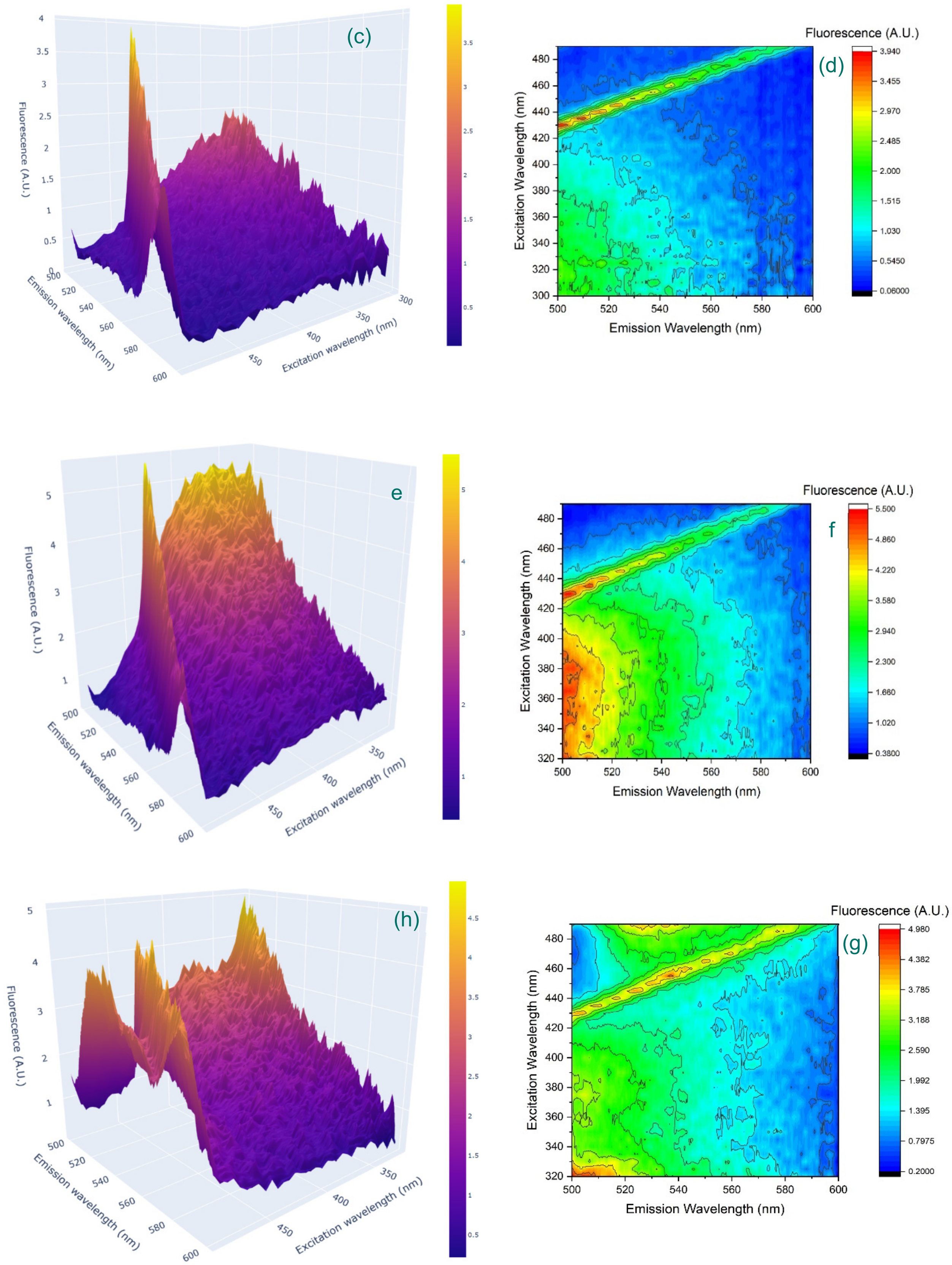

Figure S3. 3D and 2D fluorescence spectra of perfusion media, (a, b) Belzer. (c, d) Celsior. (e, f) Custodiol. (h, g) IGL1.

It is evident that the emission of the Belzer solution at 500-600 nm is 5-10 times higher than that of Celsior and Custodiol. Notably, a considerable diagonal elevation is observed in both Celsior and Custodiol, likely due to their similar compositions. However, the overall emission of Celsior and Custodiol remains very low..

Section S6. Fluorescence spectra of FMN and NADH

Depending on the excitation wavelengths, FMN's spectra exhibit varying emission intensities. Figure S4 present the 3D and 2D emission spectra of FMN at different excitation wavelengths in saline water.

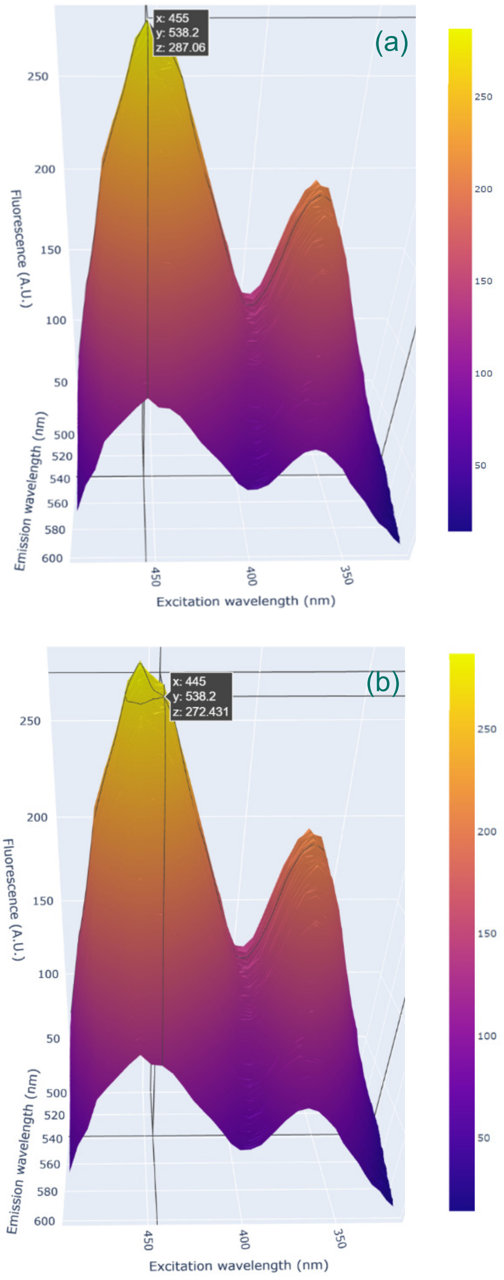

Figure S4. Indication of emission value for excitation at 455 nm (a). and 445 (b)

The maximum fluorescence peak obtained at around 538 nm by exciting at 455 nm is higher than the emission induced by excitation at 445 nm. This slight shift in excitation wavelength is expected, considering that FMN absorbs with a peak at 445 nm. Specifically, the emission peak at 538 nm measures 287 A.U. for excitation at 455 nm, compared to 272 A.U. for excitation at 445 nm. Although this minor difference may seem negligible, it could result in two distinct calibration curves. The spectra of FMN in water at higher concentrations maintain the same shape. Figure S5 show the fluorescence spectra of FMN due to excitation at 455 nm and 445 nm at varying concentrations..

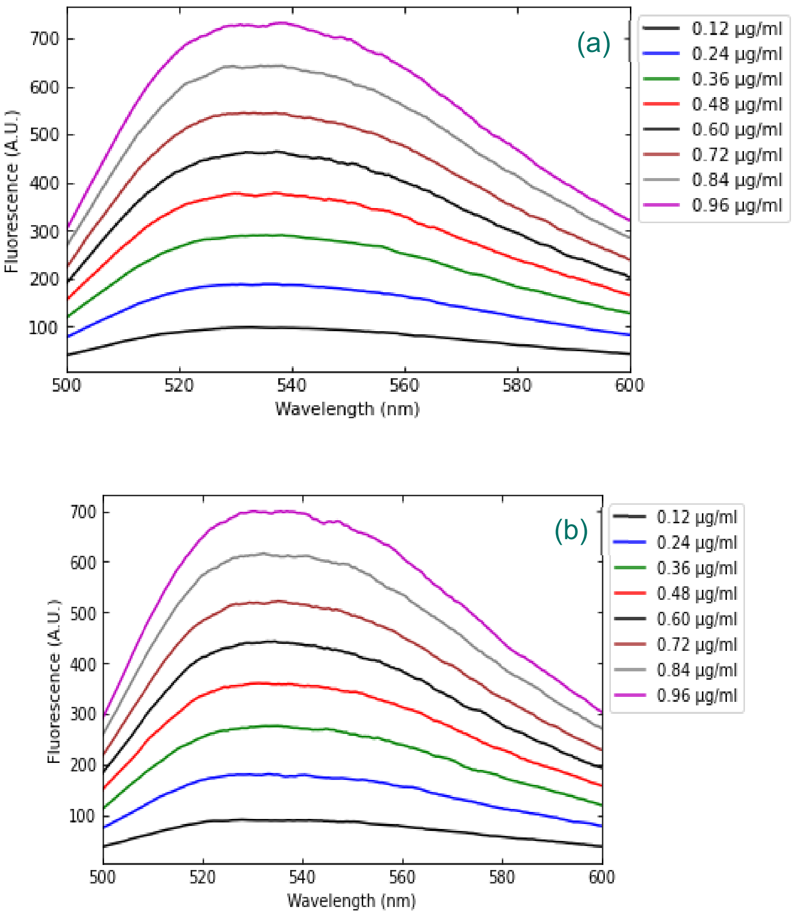

Figure S5. Spectra of FMN for excitation at 455 nm (a) and at 445 nm (b).

It is evident that the calibration curves differ based on the excitation wavelength. Figure S6 illustrates how the emissions change for a minimal concentration of 0.12 µg/mL at different excitation wavelengths.

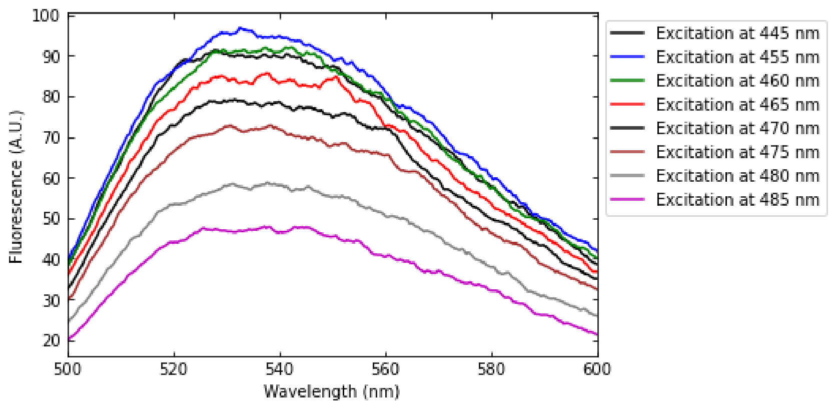

Figure S6. Spectra of FMN for different excitation wavelengths at the concentration of 0.12 μg mL^{-1}

In Figure S7 are plotted spectra of FMN in perfusion media. It appears that FMN does not interact with components for all concentration range studied.

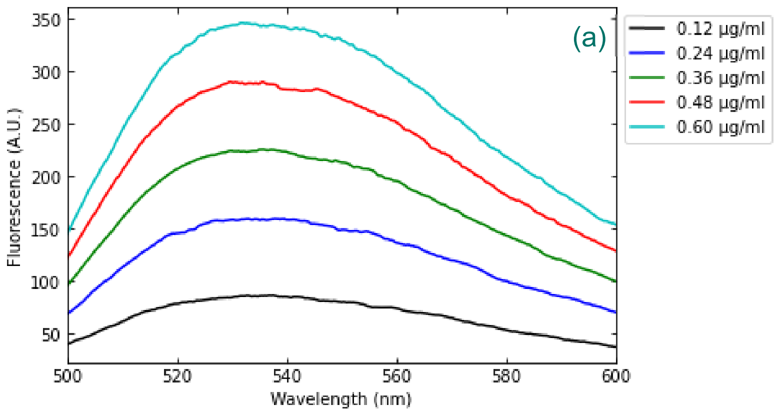

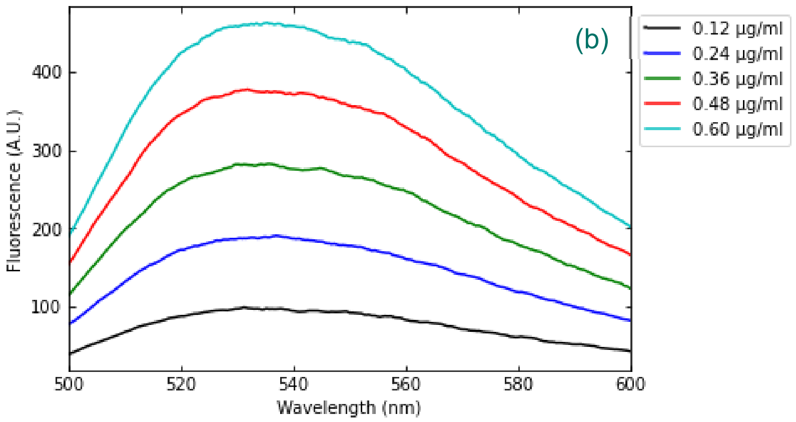

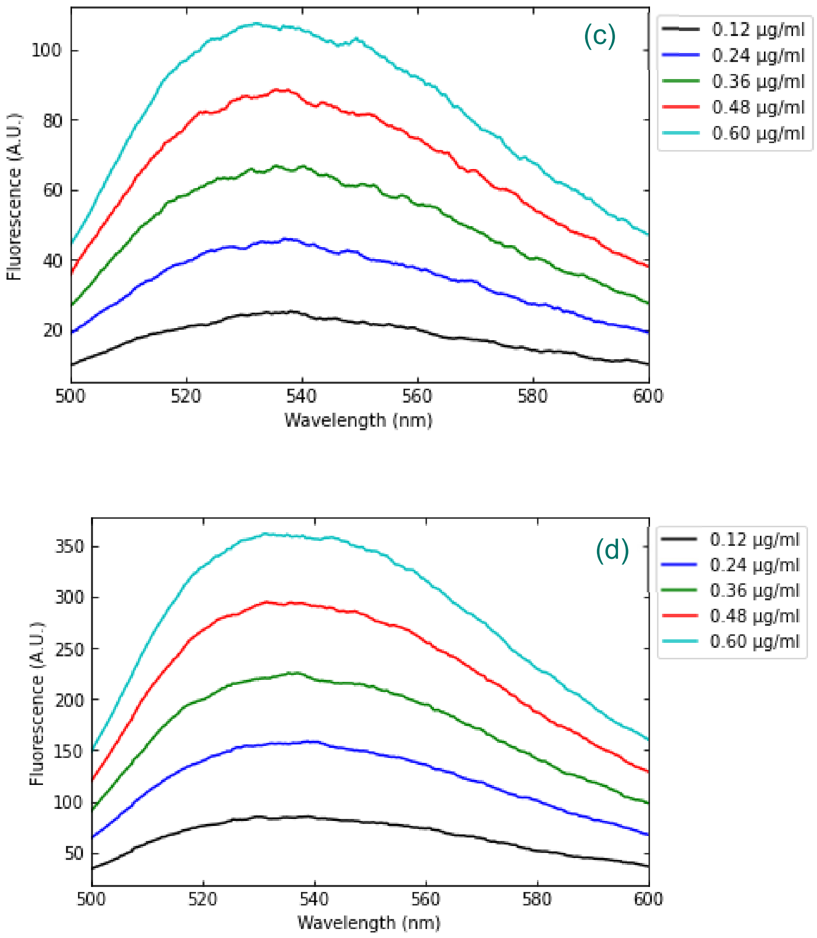

Figure S7. Spectra of FMN in different perfusion solutions. (a) In Belzer. (b) In Celsior. (c) In Custodiol. (d) In IGL1.

However, the composition of the perfusion solution affects FMN fluorescence. As shown in Figure S7, perfusion solutions have different capabilities to absorb light in the 400-490 nm range. Belzer solution absorbs light in the blue region. In water and Celsior, all available photons are absorbed by FMN because these solutions do not absorb photons in the blue region. Conversely, in the FMN-perfusion solution mixture, a portion of the photons is absorbed by the medium itself (as shown in Figure S12a for the Belzer-FMN mixture), leaving fewer photons available to excite FMN. Consequently, this reduction in the quantity of photons available to excite FMN leads to decreased FMN emission. However, the reduction of FMN fluorescence in other media like IGL1 and Custodiol could be due to the effects of the solvents, as reflected in the emission spectra represented above.

Belzer MPS perfusion solution is widely used in the field of hypothermic perfusion of kidneys and livers. Table S2b reports the fluorescence values of FMN at 538 nm for excitation at 455 nm, both in saline solution (reference values) and in Belzer MPS solution.

Table S2b. Fluorescence emission values of FMN in saline solution and in Belzer MPS for different concentrations.

Concentration of FMN µg mL^{-1}	*FMN Fluorescence in Saline Solution (A.U.)*	*FMN Fluorescence in Belzer MPS (A.U.)*
0.1	83.025	81.415
0.2	159.8	134.83
0.3	236.575	188.245
0.4	313.35	241.66
0.5	390.125	295.075
0.6	466.9	348.49
0.7	543.675	401.905
0.8	620.45	455.32
0.9	697.225	508.735
1	774	562.15
1.1	850.775	615.565
1.2	927.55	668.98

Although the fluorescence of FMN in Belzer MPS is lower than in saline solution, the data clearly show that the fluorescence signal is still acceptable. It is observed that the fluorescence of FMN at a concentration of 0.1 µg mL^{-1} is identical in both saline solution and Belzer MPS. However, a divergence in values is noted at concentrations of 0.3 µg mL^{-1} and 0.5 µg mL^{-1}, where the fluorescence of FMN in Belzer is 20% and 25% lower, respectively, compared to that in saline solution. At a concentration of 1.2 µg mL^{-1}, it is predicted that the fluorescence of FMN in Belzer MPS will be 27% lower. Nevertheless, this value remains appreciable, especially when compared to the low fluorescence observed in Custodiol solution. These data clearly demonstrate that FMN is as easily detectable in Belzer MPS as in saline solution. Similar observations are still valid of IGL-1 solutions, although the last one is less used during hypothermic machine perfusion.

In Table S3a and S3b we report the standard deviation of errors bars shown in Figures 9 and 11

Table S3a. Standard deviations of the replicate measurements shown in Figure 9.

**Concentration (µg/mL)**	**Saline sol. Error**	**Belzer Error**	**Celsior Error**	**Custodiol Error**	**IGL1 Error**
**0.12**	3.567	0.910	0.989	0.683	1.286
**0.24**	1.582	1.720	0.988	0.292	0.767
**0.36**	4.119	1.907	2.587	1.953	1.159
**0.48**	2.310	1.767	1.183	0.145	2.358

**0.60**	0.397	2.889	3.658	0.803	1.792

Table S3b. Standard deviation values of replicate measurements shown in Figure 11.

Concentration (µg/mL)	Std Dev at 340 nm	Std Dev at 360 nm
20	1.839	4.341
40	8.115	4.596
60	8.091	3.030
80	12.992	4.214
100	4.863	6.663
120	3.977	6.742
140	0.942	5.575
160	4.493	8.530
180	2.683	13.902
200	9.861	17.203

Regarding NADH fluorescence, it was observed that its fluorescence shape changes based on concentration. At higher concentrations, a second peak appears in the deep ultraviolet region, as shown in Figure S8. To provide a precise comparison, Table S4 reports values of NADH emission for different excitation wavelengths at various concentrations.

Table S4. Values of emissions for different excitation wavelength in various concentrations

	20 µg/ml	*100 µg/ml*	*160 µg/ml*	*200 µg/ml*
Emission at 465 nm for 340 nm of excitation	**306.306 A.U.**	869.509 A.U.	904.822 A.U.	864.662 A.U.
Emission at 465 nm for 350 nm of excitation	301.281 A.U.	**890.695 A.U.**	946.755 A.U.	921.188 A.U.
Emission at 465 nm for 360 nm of excitation	261.818 A.U.	854.245 A.U.	**963.966 A.U.**	995.762 A.U.
Emission at 465 nm for 365 nm of excitation	226.643 A.U.	791.363 A.U.	933.061 A.U.	**1007.91 A.U.**

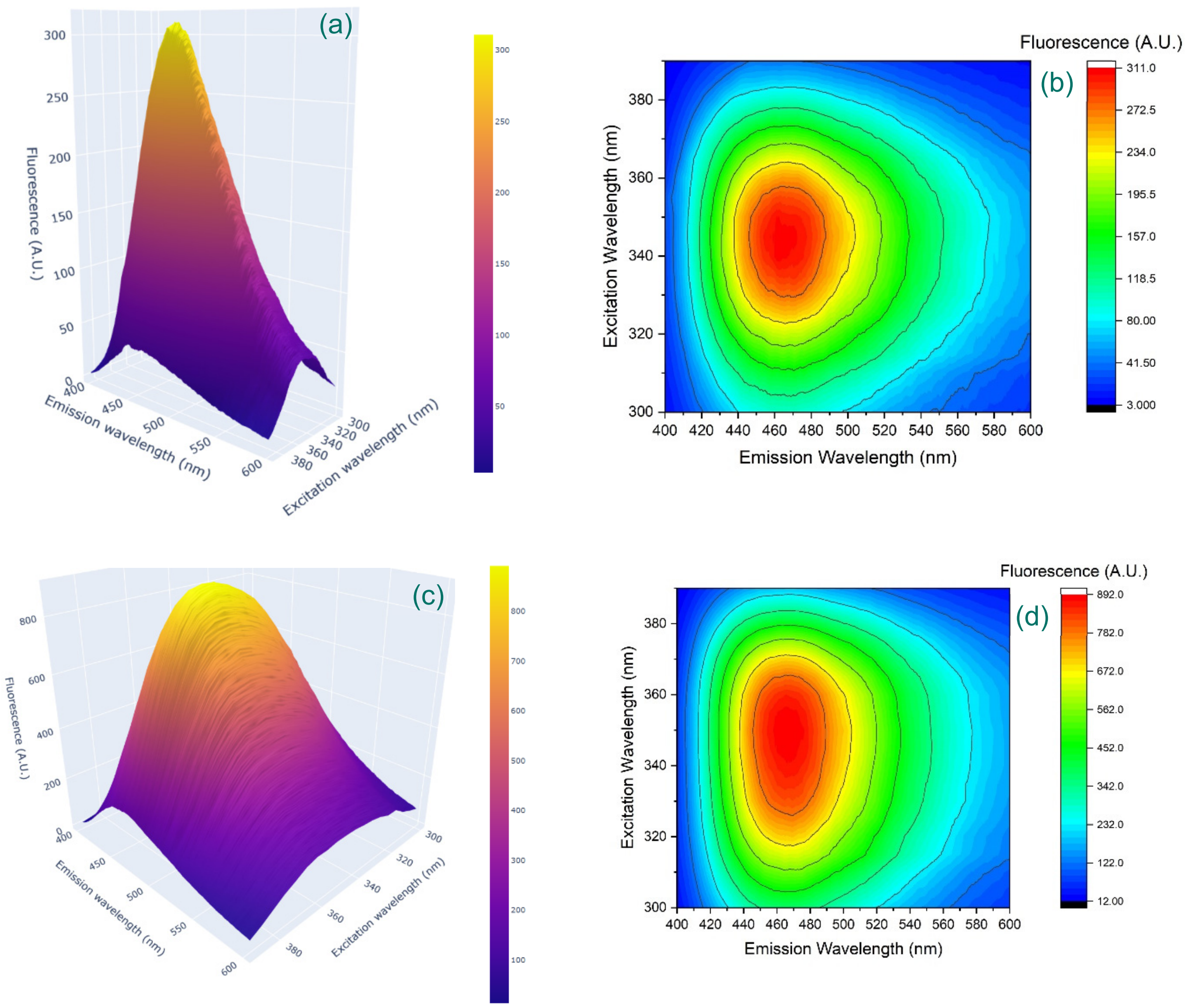
(a)
Fluorescence (A.U.)
300
250
200
150
100
50
0
Emission wavelength (nm)
400
450
500
550
600
Excitation wavelength (nm)
300
320
340
360
380
(b)
Fluorescence (A.U.)
311.0
272.5
234.0
195.5
157.0
118.5
80.00
41.50
3.000
Excitation Wavelength (nm)
Emission Wavelength (nm)
400 420 440 460 480 500 520 540 560 580 600
(c)
Fluorescence (A.U.)
800
600
400
200
0
Emission wavelength (nm)
Excitation wavelength (nm)
(d)
Fluorescence (A.U.)
892.0
782.0
672.0
562.0
452.0
342.0
232.0
122.0
12.00
Excitation Wavelength (nm)
Emission Wavelength (nm)

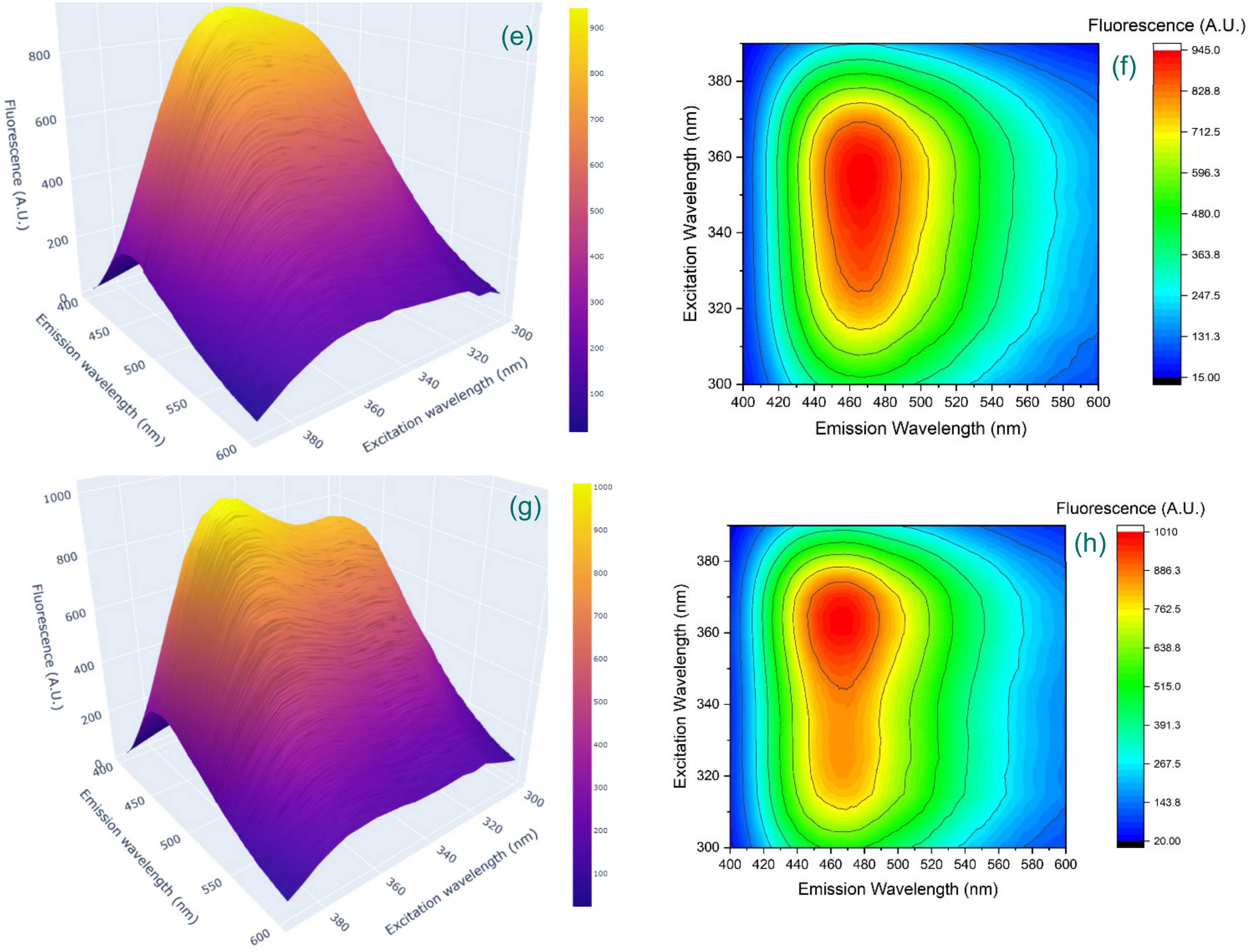

Figure S8. 3D and 2D fluorescence spectra of NADH in water at different concentration. (a, b) 20 µg mL^{-1}. (c, d) 100 µg mL^{-1}. (e, f) 140 µg mL^{-1}. (h, g) 200 µg mL^{-1}.

It's clear that increasing concentration causes a shift in emission peak at 465 nm. This peculiarity makes it challenging the choice of optimal excitation wavelength.

Section S7. Mixture NADH + FMN

To study the 3D spectra of FMN and NADH in perfusion solutions, their concentrations were analyzed within the same range as when studied individually, varying both FMN and NADH. From a clinical perspective, the relationship between FMN and NADH release is not well understood and requires further investigation. In this study, fluorescence was evaluated starting from an FMN concentration of 0.12 µg/mL and an NADH concentration of 20 µg/mL, increasing both simultaneously. The concentration matrix, shown in Table S5, represents all possible combinations of FMN and NADH concentrations. The spectrophotometric analysis focused along the diagonal of the concentration matrix, considering it less useful to examine other ratios, as simultaneous variation

of NADH and FMN in the perfusate is deemed more plausible.

Table S5. Concentration matrix. Along with raw FMN concentration varies, along with the column the NADH concentration varies.

**0.12;20**	0.24;20	0.36;20	0.48;20	0.60;20	0.72;20	0.84;20	0.96;20	1.08;20	1.20;20
0.12;40	**0.24;40**	0.36;40	0.48;40	0.60;40	0.72;40	0.84;40	0.96;40	1.08;40	1.20;40
0.12;60	0.24;60	**0.36;60**	0.48;60	0.60;60	0.72;60	0.84;60	0.96;60	1.08;60	1.20;60
0.12;80	0.24;80	0.36;80	**0.48;80**	0.60;80	0.72;80	0.84;80	0.96;80	1.08;80	1.20;80
0.12;100	0.24;100	0.36;100	0.48;100	**0.60;100**	0.72;100	0.84;100	0.96;100	1.08;100	1.20;100
0.12;120	0.24;120	0.36;120	0.48;120	0.60;120	**0.72;120**	0.84;120	0.96;120	1.08;120	1.20;120
0.12;140	0.24;140	0.36;140	0.48;140	0.60;140	0.72;140	**0.84;140**	0.96;140	1.08;140	1.20;140
0.12;160	0.24;160	0.36;160	0.48;160	0.60;160	0.72;160	0.84;160	**0.96;160**	1.08;160	1.20;160
0.12;180	0.24;180	0.36;180	0.48;180	0.60;180	0.72;180	0.84;180	0.96;180	**1.08;180**	1.20;180
0.12;200	0.24;200	0.36;200	0.48;200	0.60;200	0.72;200	0.84;200	0.96;200	1.08;200	**1.20;200**

Given that the FMN concentration is approximately 100 times lower than the NADH concentration, FMN fluorescence contributes weakly but detectably to the 3D spectra. As the concentration of FMN in the FMN+NADH mixture increases, the influence of FMN fluorescence becomes more pronounced due to its absorption in ultraviolet light, particularly in the 340-380 nm range. From Figure S9 and particularly from Figure S10, a notable peak arises in the 500-530 nm region due to excitation at 340-360 nm. This suggests the possibility of mutual excitation between NADH and FMN.

NADH emits light in the blue region, where FMN absorbs. Due to the higher concentration of NADH, the light emission of NADH in the blue region upon excitation at 360-370 nm could have excited FMN, causing the peak in the yellow region to rise. This hypothesis is supported by the higher concentration of NADH relative to FMN. It is plausible that a significant portion of photons are absorbed by NADH molecules, with FMN emission resulting from NADH fluorescence. Figure S10 present 2D heat map spectra at the same concentration to reinforce this explanation.

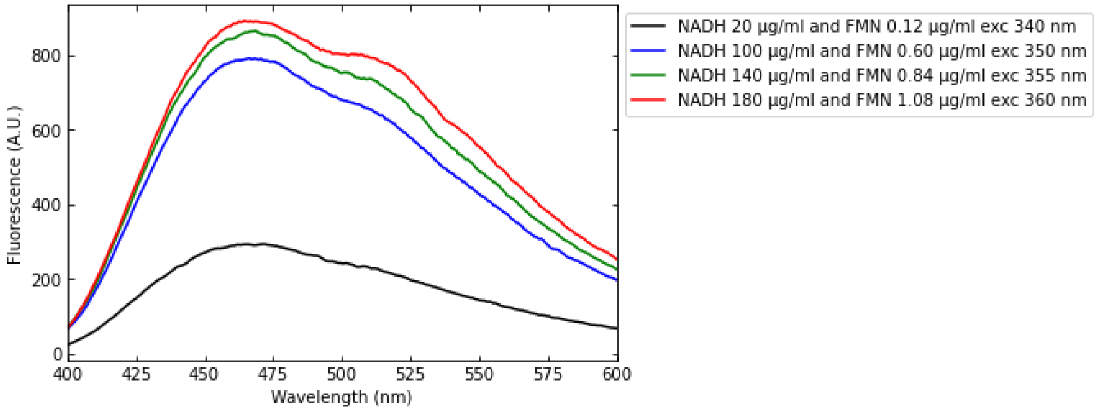

Figure S9. Spectra of the mixture NADH+FMN in water for different concentrations and excitation wavelengths.

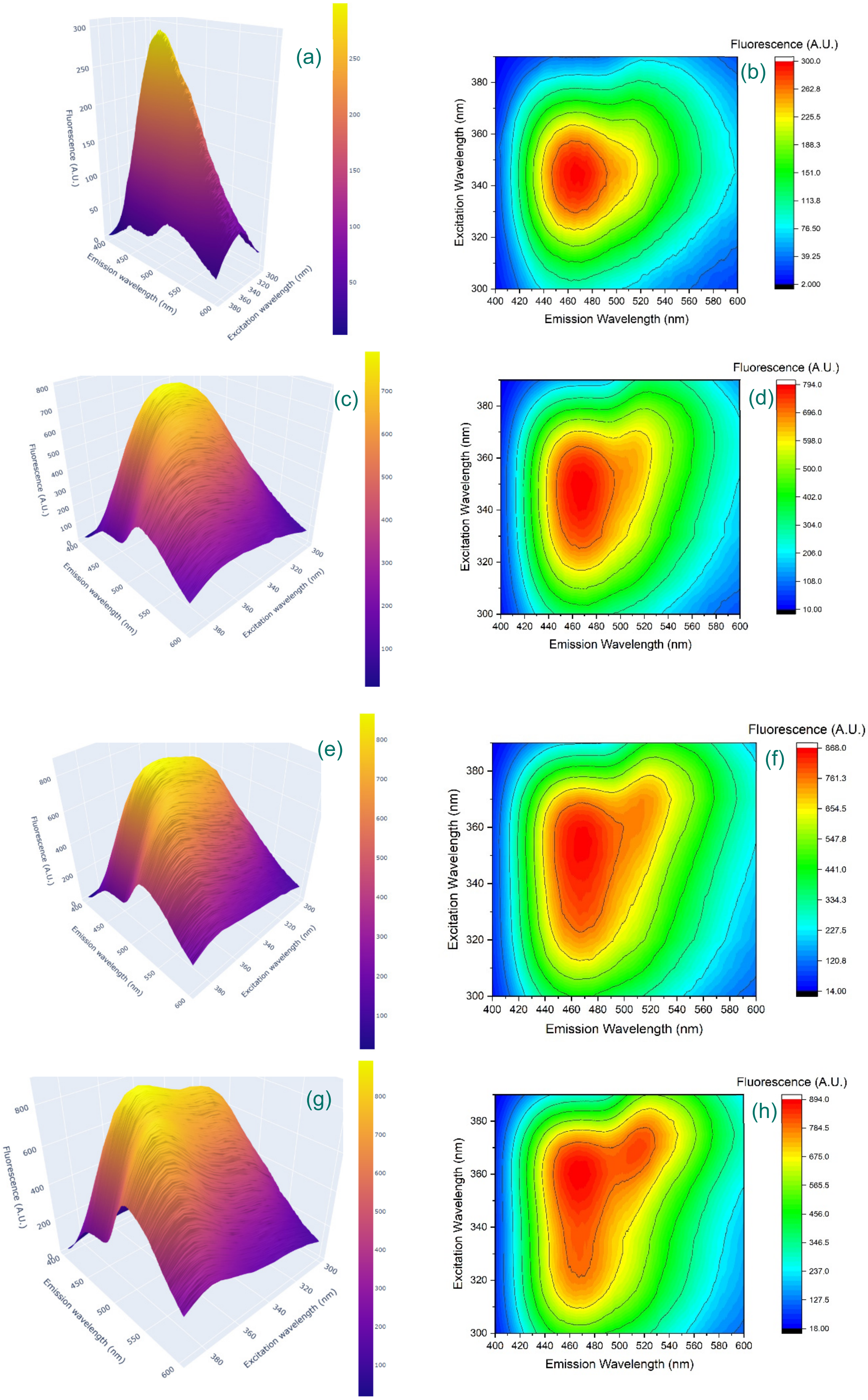

Figure S10. 3D and 2D fluorescence spectra of FMN+NADH mixture in water at different

concentration for excitation in range 300-390 nm and fluorescence in range 400-600 nm. (a, b) FMN 0.12 μg mL^{-1}, NADH 20 μg mL^{-1}. (c, d) FMN 0.60 μg mL^{-1}, NADH 100 μg mL^{-1}. (e, f) FMN 0.84 μg mL^{-1}, NADH 140 μg mL^{-1}. (h, g) FMN 1.20 μg mL^{-1}, NADH 200 μg mL^{-1}.

Figure S10d, S10f, and S10h confirm that the fluorescence generated by FMN is primarily due to the emission by NADH. Specifically Figure S10a, which plots the spectrum of 0.12 μg/mL FMN and 20 μg/mL NADH, appears very similar to the NADH spectrum depicted in Figure S8a. As the concentration of NADH increases, the capability of exciting FMN through NADH emission also increases. However, exciting the FMN+NADH mixture in the 340-360 nm range allows for the observation of NADH fluorescence at 465 nm without significant interference from FMN fluorescence.

Figure S11 show the 3D spectra of the same mixture but excited in the 300-490 nm range, displaying fluorescence in the 500-600 nm range .

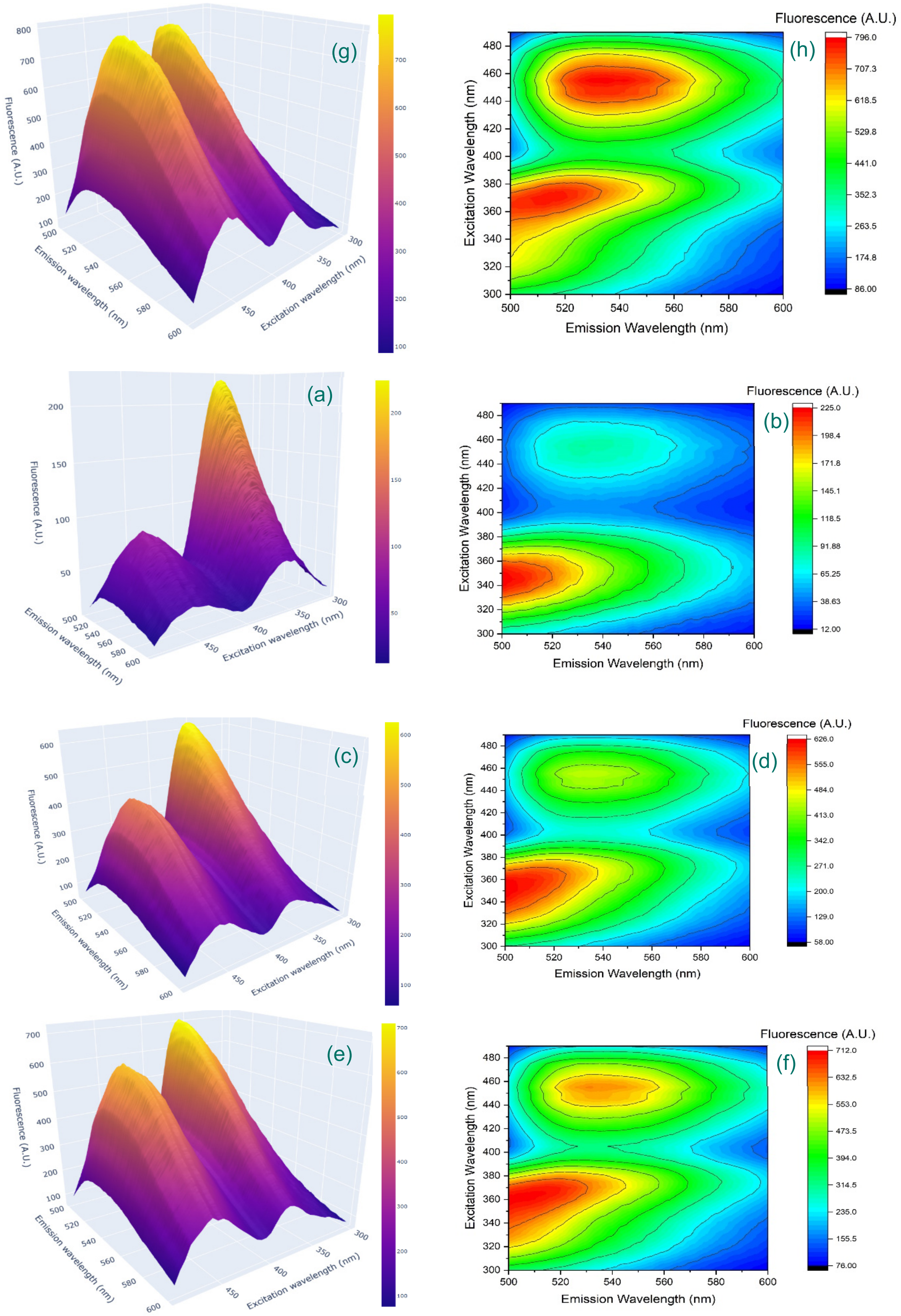
(g)
(h)
(a)
(b)
(c)
(d)
(e)
(f)
Fluorescence (A.U.)
Emission wavelength (nm)
Excitation wavelength (nm)
Excitation Wavelength (nm)
Emission Wavelength (nm)
796.0
707.3
618.5
529.8
441.0
352.3
263.5
174.8
86.00
225.0
198.4
171.8
145.1
118.5
91.88
65.25
38.63
12.00
626.0
555.0
484.0
413.0
342.0
271.0
200.0
129.0
58.00
712.0
632.5
553.0
473.5
394.0
314.5
235.0
155.5
76.00

Figure S11. 3D and 2D fluorescence spectra of FMN+NADH mixture in water at different concentration for excitation in range 300-490 nm and fluorescence in range 500-600 nm. (a, b) FMN 0.12 μg mL^{-1}, NADH 20 μg mL^{-1}. (c, d) FMN 0.60 μg mL^{-1}, NADH 100 μg mL^{-1}. (e, f) FMN 0.84 μg mL^{-1}, NADH 140 μg mL^{-1}. (h, g) FMN 1.20 μg mL^{-1}, NADH 200 μg mL^{-1}.

It appears straightforward that FMN fluorescence is caused by NADH fluorescence when excitation wavelengths range from 300 to 390 nm in the ultraviolet region, as shown in Figure S11e, S11f, S11g, and S11h. The higher peak at 340-365 nm is indeed due to the presence of NADH. Essentially, from a spectrophotometric point of view, NADH is the predominant species in this mixture.

However, when the mixture is excited in the blue region, such as at 455 nm, FMN fluorescence becomes evident. When excited at 455 nm, only FMN fluorescence is observed because NADH absorbs up to 390 nm. Conversely, when excited in the 340-360 nm region, NADH fluorescence increases. Given that the concentration of NADH is 100 times greater than that of FMN, the photons will be largely absorbed by the NADH molecules. Indeed, in all of Figure S12, the emission peak at 500 nm for excitation at 340-360 nm is due primarily to NADH, with negligible contribution from FMN. Increasing the concentration of FMN also reveals the characteristic fluorescence peak of FMN. The 2D fluorescence spectra in Figure S11 are irregular due to the mutual interaction between NADH and FMN.

It is important to highlight that the calibration curve of FMN in the FMN+NADH mixture in water remains linear in fluorescence. Figure S12 present the calibration curve of FMN for excitation at 455 nm and of NADH for excitation at 340 nm and 360 nm in the FMN+NADH mixture in water.

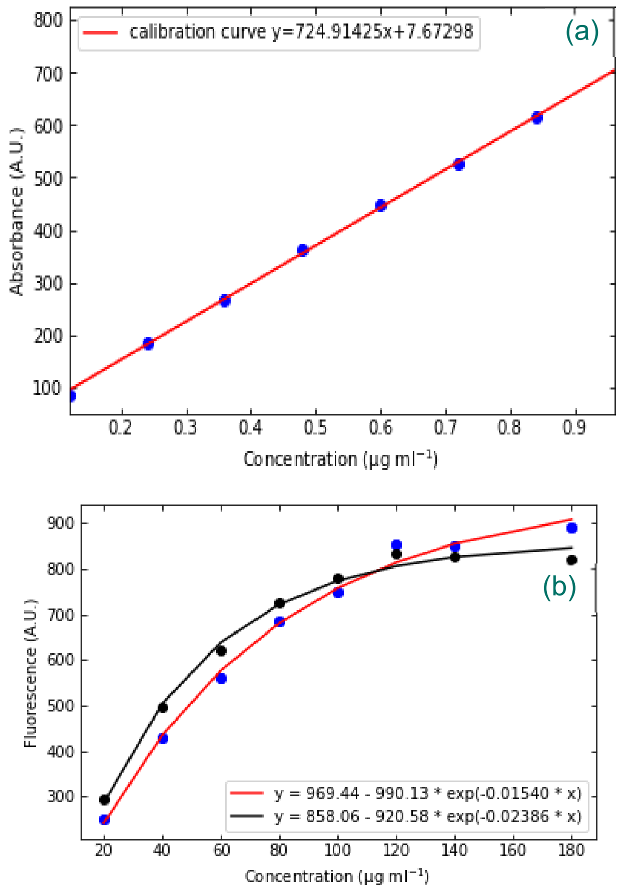

Figure S12. (a) Calibration curve for FMN in mixture FMN+NADH. (b) Calibration curve for NADH in mixture FMN+NADH.

It is possible to verify that the calibration curves for FMN in water and in the FMN+NADH mixture in water are the same. The differences between these two curves could be attributed to experimental errors. The previous observations about the effects of perfusion solutions on FMN and NADH remain valid in the FMN+NADH mixture system.

The same reasoning applies to NADH in water and in the FMN+NADH mixture. For NADH, the differences in calibration curves between NADH in water and the NADH+FMN mixture are more evident, likely due to experimental errors.
